# Bi-specific T-cell engagers (BiTEs) in prostate cancer and strategies to enhance development: hope for a BiTE-r future

**DOI:** 10.3389/fphar.2024.1399802

**Published:** 2024-05-30

**Authors:** Harriet Lampe, Laura Tam, Aaron R. Hansen

**Affiliations:** Department of Medical Oncology, Division of Cancer Care Services, Princess Alexandra Hospital, Metro South Health Service, Queensland Health, Brisbane, QLD, Australia

**Keywords:** prostate cancer, metastatic castrate resistant prostate cancer, novel immunotherapies, bi-specific T-cell engager therapy, T-cell engager therapy, bi-specific antibody therapy, BiTE

## Abstract

Metastatic castrate resistant prostate cancer (mCRPC) continues to have poor survival rates due to limited treatment options. Bi-specific T cell engagers (BiTEs) are a promising class of novel immunotherapies with demonstrated success in haematological malignancies and melanoma. BiTEs developed for tumour associated antigens in prostate cancer have entered clinical testing. These trials have been hampered by high rates of treatment related adverse events, minimal or transient anti-tumour efficacy and generation of high titres of anti-drug antibodies. This paper aims to analyse the challenges faced by the different BiTE therapy constructs and the mCRPC tumour microenvironment that result in therapeutic resistance and identify possible strategies to overcome these issues.

## 1 Introduction

Globally, prostate cancer is the second most common cancer diagnosed amongst men and accounts for the fifth most common cause of malignancy-related deaths (Sung et al., 2021). Androgen deprivation therapy (ADT) has long remained the backbone of treatment of locally advanced or metastatic prostate cancer. The past 2 decades have seen multiple advancements in prostate cancer treatment, with demonstration of improved overall survival in metastatic castrate sensitive prostate cancer (mCSPC) with the introduction of androgen receptor signal inhibitor (ARSi) therapy, docetaxel chemotherapy and external radiation therapy, and in metastatic castrate resistant prostate cancer (mCRPC) with ARSis, a broader range of chemotherapy agents, and Radium-223 radionucleotide treatment (Petrylak et al., 2004; de Bono et al., 2011; Parker et al., 2013; Beer et al., 2014; James et al., 2016; Fizazi et al., 2017; Kyriakopoulos et al., 2018; Armstrong et al., 2019; Chi et al., 2019; Fizazi et al., 2022; Smith et al., 2022). The PSMA-targeted Lutetium-177 radionucleotide therapy has also shown survival benefit in mCRPC patients in the post-chemotherapy setting (Sartor et al., 2021). Additionally, for approximately 20% of men with mCRPC who harbour a homologous recombination defect (HRD), poly-ADP-ribose-polymerase (PARP) inhibitors such as olaparib and rucaparib have demonstrated better survival outcomes and are approved therapies in many countries (Hussain et al., 2020; Fizazi et al., 2023). Despite these significant advancements, overall survival rates amongst those with mCRPC remains low at only 34% at 5 years, thereby necessitating a need to develop better therapeutic options (SEER Explorer, 2023). BiTEs may represent a drug class that has the potential to improve clinical outcomes for prostate cancer patients and provide them with hope for a “brighter” future.

### 1.1 Immunotherapies in prostate cancer

Immune checkpoint inhibitors (ICI) have revolutionised the management of a plethora of malignancies in the past decade (Robert, 2020). However, while their use in mCRPC has shown a marginal benefit in a small proportion of prostate cancer patients with mismatch repair (MMR) deficiency mutations, their use in the majority of mCRPC patients has been limited with a number of negative trials including KEYNOTE-641 and the KEYLYNK-010 trial (Kwon et al., 2014; Robinson et al., 2015; Kazandjian et al., 2016; Beer et al., 2017; Hansen et al., 2018; Antonarakis et al., 2019; Sharma et al., 2019). Earlier research in cancer vaccines have shown some success in the prostate cancer landscape. Sipuleucal-T, a dendritic cell-based vaccine which acts via a recombinant protein of granulocyte macrophage colony stimulating factor (GM-CSF) and prostatic acid phosphatase (PAP) to facilitate maturation of PAP-expressing antigen presentation cells, with subsequent T cell activation and PAP-expressing prostate cell killing, was approved by the US Food and Drug Administration (FDA) in 2010 for minimally symptomatic mCRPC. Although it demonstrated an overall survival (OS) benefit, particularly in a sub-population with reduced metastatic burden, and has acted as an important proof of concept for immunotherapies outside of ICIs in prostate cancer treatment, sipuleucal-T has failed to be incorporated into routine treatment for mCRPC due to doubts about limited clinical benefit (Kantoff et al., 2010). These modest results have driven research into other facets of immunotherapy such as T-cell engager (TCE) therapies, which aim to directly crosslink T-cells with tumour associated antigens (TAAs), thus driving localised cancer cell specific cytotoxicity, cytokine release, and downstream activation of a B-cell polyclonal humoral response (Zhou et al., 2021). TCE therapies include chimeric antigen-receptor-modified (CAR) T cells, in which patients’ own T-cells are genetically engineered *ex vivo* to express a chimeric T-cell receptor targeted at the desired TAA, and bi-specific T-cell engagers (BiTEs), antibody-based molecules offering a promising “off the shelf” option for achieving cross-linking of cancer and T-cells, which we shall examine in further detail.

### 1.2 BiTE therapies in cancer

BiTEs are monoclonal antibody (mAb) based molecules comprised of at least two conjoined antibody components which have respective specificity for a TAA of choice, and for an immune cell component, typically the conserved portion of the T-cell receptor, CD3, connected by a flexible linker moiety (Riethmüller, 2012). The mechanism of action of BiTEs involves activation of cytotoxic T-cells independent of the co-stimulation pathway, resulting in robust killing of cancer cells which has been demonstrated *in vitro* to have efficacy 100–10,000 fold greater than that of mAbs (Wolf et al., 2005).

BiTEs can be produced through chemical cross-linking of two antibody fragments, or via fusion of two different monoclonal antibody producing cell lines (so called “quadromas”) with subsequent purification of the desired protein outcome (Schaefer et al., 2011). Initial inefficiencies in BiTE production concerning high proportions of incorrect heterodimer products have been addressed with multiple strategies to improve correct heterodimerisation. These have included heavy chain “knobs in holes” alterations (Xu et al., 2015), “crossover” of light and heavy chain combinations within one Fab arm of a bispecific IgG antibody to reliably produce a single desired heterodimer (CrossMabs; (Klein et al., 2019)), and dual affinity re-targeting proteins (DARTs), which favour stable dimerization due to exchange of variable heavy and light chains between two scFv components (Johnson et al., 2010). While the original BiTE molecules comprised two single chain variable fragments (scFv) from two different mAbs connected by a flexible peptide linker, further drug development research has generated an array of structural variations conferring benefits of more efficient production, increased half-life and improved target binding (Suurs et al., 2019). Blinatumomab, an anti-CD3-CD19 BiTE, was the first in its class to be approved by the FDA in 2014 for the treatment of Philadelphia chromosome negative relapsed or refractory B-cell acute lymphoblastic leukaemia (Kantarjian et al., 2017). In solid tumours, tebentafusp, which targets gp100 and CD3, has demonstrated improved survival outcomes for patients with uveal melanoma (Nathan et al., 2021). While there are no other approved solid tumour BiTEs to date, work is currently underway in a variety of cancer histologies to further their development.

### 1.3 BiTEs in prostate cancer

The use of BiTE therapies in prostate cancer has been trialled in several studies using different structures and TAA targets (See Table 1 for a complete list of finalised and ongoing trials). The first trial of a BiTE therapy in prostate cancer involved pasotuxizumab, an anti-CD3 and anti-prostate specific membrane antigen (PSMA) construct, administered either via subcutaneous injection or continuous intravenous infusion which produced a 54.9% reduction in PSA in the highest dose cohort, but was associated with 81% rate of Grade 3 or 4 treatment related adverse events (tr-AEs; (Hummel et al., 2021)). The trial was terminated in favour of acapatamab, an anti-CD3, anti-PSMA molecule with an IgG crystallisable fragment (Fc) to extend serum half-life. Unfortunately, while the PSA response of a 50% reduction (PSA50) was 34.3%, the rate of grade 3/4 tr-AEs was >50% and consequently acapatamab was not planned for further development (Ben et al., 2020). Subsequent studies have assessed alternative TAAs, including prostate stem cell antigen (PSCA), human kallikrein 2 (KLK2), delta-like protein 3 (DLL-3) and six transmembrane epithelial antigen of prostate 1 (STEAP-1). These studies have also explored a variety of alternative BiTE structures—addition of HLE or immune-interacting domains, incorporation of more complete antibody structures and differences in linker molecules. However, to date the vast majority have failed to move past Phase 1 clinical trials, displaying various combinations of prohibitive side effect profiles, limited anti-tumour efficacy and a high rate of anti-drug antibodies, amongst other issues (Simão et al., 2023). Given the increasing interest in BiTE therapies in prostate cancer, we will explore some of the factors impacting both safety and efficacy associated with this class of drugs.

**TABLE 1:** Summary of clinical trials evaluating BiTE therapies in prostate cancer to date.

Drug	Phase	Structure	Population	Route and dose	Treatment related adverse events	Anti-drug antibodies	Anti-tumour efficacy	Trial outcome	Clinical trial
Pasotuxizumab (AMG 212, BAY2010112)	1	Anti-CD3 and anti-PSMA (sequences not specified) (Lutterbuese et al., 2011)	N = 47 mCRPC refractory to ≥1 taxane regimen and abiraterone or enzalutamide, on continuous ADT, ECOG 0–2	2 arms: Daily s.c. injection, 21 days cycles, dose cohorts ranging from 0.5 µg to 172 µg. Or c.i.v. infusion, 6 weeks cycles with 1 week break, dose cohorts of 20, 40 and 80 µg/day cohorts	100% experienced AEs any grade (majority CRS and fatigue). 81% Grade 3–4 (44% lymphopaenia, 44% infections)	Detected in 100% of s.c. arm, nil change with dexamethasone pre-medication. 93% were neutralising. ADAs associated with reduced drug serum concentration however limited data available. Titres did not correlated with AEs or drug dose received	Pre-clinical: EC50 3.4–6.7 ng/mL in PSMA human cell culture	Prematurely terminated—in favour of AMG 160. MTD not established	NCT01723475
						0% in c.i.v.arm. (Hweixian et al., 2023)	Regression of prostate cancer xenografts in mice post s.c. injection (Friedrich et al., 2012)		
							Clinical: S.c. arm −24.7% PSA decline. C.i.v. arm −22.0, −37.7% and −54.9% in 20, 40 and 80 µg/day dosing cohorts (Hummel et al., 2021)		
Acapatamab (AMG 160) ± pembrolizumab	1	Anti-CD3 and anti-PSMA and IgG Fc active fragment (HLE) (Sequences not specified) (Deegen et al., 2021)	N = 43 (monotherapy AMG 160). mCRPC refractory to 1–2 taxane regimens and abiraterone or enzalutamide, on continuous ADT, ECOG 0–1	i.v. dose ranges 0.003–0.9 mg fortnightly	95.3% any grade. >50% Grade 3–4. 31.3% Grade 3 CRS.	Neutralizing ADAs were detected, limited data available	Pre-clinical: EC50 6–42 pmol/L at 42 h in PSMA PC cell lines	Completed without public release of final results. Preliminary data released	NCT03792841
							Clinical: PSA50 in 34.3%		
							PR in 13.3%, SD in 53.3% (Ben et al., 2020; Deegen et al., 2021)		
Acapatamab ± (AMG 404 or enzalutamide or abiraterone) or AMG 404	1	As for AMG 160	N = 65 mCRPC with continuous ADT.	I.v. dosing of acapatamab (Subudhi et al., 2021)	Not reported	Not reported	Not reported	Terminated for business decision, nil further information released	NCT04631601
Solitomab (AMG 110, MT 110)	1	Anti-CD3 and anti-EpCAM (Sequences not specified)	N = 65 (3 with mCRPC). Locally advanced, recurrent or metastatic solid tumours known to express EpCAM.	c.i.v. dosing, protocol not specified	95% Grade ≥3	Not assessed	One unconfirmed PR (Kebenko et al., 2018)	Completed	NCT00635596
APVO414 (ES414, MOR209)	1	Anti-CD3 and anti-PSMA and passive IgG Fc region (HLE) (ADAPTIR^®^) (Sequences not specified) (Hernandez-Hoyos et al., 2016)	N = 18. CRPC, refractory to abiraterone or enzalutamide, ECOG 0–1, NEPC excluded	2 arms: Weekly i.v. dosing and c.i.v	Not reported	58% of weekly i.v. dosing cohort developed ADAs with very high titres which reduced drug exposure	Not reported	Completed without public release of final results	NCT02262910
						50% of c.i.v. cohort developed ADAs of lower titres. (Author Anynomus, 2017)			
HPN424	1/2a	Anti-CD3 and anti-PSMA and anti-albumin (HLE) (TriTAC^®^) (Sequences not specified)	N = 110 mCRPC, received ≥2 prior systemic therapies, ongoing ADT	Weekly i.v. dose ranging 1.3–300 ng/kg with step—up dosing regimen	40% Grade ≥3 (18% AST elevation, 11% anaemia, 11% ALT elevation). 63% CRS any grade, 4% CRS Grade ≥3	Not assessed	21% any PSA reduction. 2 PSA30 and 3 PSA50 responses. 1 radiologic PR. (Bono et al., 2021)	Active, no longer recruiting. Challenging safety profile precluding further research. (Author Anynomus, 2022)	NCT03577028
JNJ-63898081 (JNJ-081)	1	Anti-CD3 and anti-PSMA and IgG4 Fc chain (HLE) (Sequences not specified) (DuoBody^®^)	N = 40 mCRPC or mRCC, refractory to ≥1 prior line of therapy, ECOG 0–1	3 arms: i.v. weekly dose ranging 0.1–3.0 μg/kg, s.c. weekly dose ranging 3–30 μg/kg, or s.c. escalation protocol with target doses ranging 30–60 μg/kg	43.6% Grade ≥3. CRS any grade 66.7%. 84.6% any grade injection/infusion reaction	63% in s.c. groups, 16.7% in i.v. group. In s.c. dosing, ADA associated with decreased drug exposure. 1 case of reversal of PSA30 in setting of developing ADAs	Transient PSA reduction in s.c. dosing >30 μg/kg. 2 subjects achieved PSA50 reduction. Cytokine release was more variable and overall reduced when compared with i.v. dosing. (Lim et al., 2022)	Completed	NCT03926013
CCW702	1	Anti-CD3 (UCHT1 construct) and anti-PSMA DUPA molecule) with triazole linker (Lee et al., 2021)	N = 22 mCRPC refractory to at least one novel androgen receptor targeted therapy, ECOG 0–1 (Markowski et al., 2021)	Daily s.c., dosing not specified	Not reported	Not reported	Not reported	Terminated early 2023 for business decision, nil further information publicly available	NCT04077021
CC-1	1	Anti-CD3 and anti-PSMA (10B3, proprietary) and IgG1 Fc (IgGsc format) (HLE). (Zekri et al., 2021)	N = 66, CRPC refractory to ≥3 lines of therapy, ECOG 0–2	c.i.v., target dose 826 µg	88% CRS (all Grade 1–2) despite prophylactic tocilizumab. 46% hypertension any grade	Not assessed	All except 1 subject had PSA reduction, not quantified (Hackenbruch et al., 2023)	Recruiting, due primary completion December 2024	NCT04104607
CC-1	1	Anti-CD3 (UCHT1 construct) and anti-PSMA (10B3 construct, proprietary) and IgG1 Fc (IgGsc format) (HLE). (Zekri et al., 2021)	N = 56 (Estimated), CRPC with biochemical recurrence post ≥1 line therapy, with low risk of rapid disease progression	3 hours i.v. infusion target dose ranging 78–600 µg with step-up dosing. (Hackenbruch et al., 2023)	Pending	Pending	Pending	Recruiting, due primary completion December 2024	NCT05646550
GEM3PSCA	1	Anti-CD3 and anti-PSCA (Sequences not specified)	N = 23, PSCA positive cancer (Renal, prostate, NSCLC) refractory to standard treatments, ECOG 0–2	1 week c.i.v. dosing (Clinicaltrials, 2019)	Not reported	Not reported	Not reported	Terminated for business decision, nil further information publicly available	NCT03927573
JNJ-78278343	1	Anti-CD3 and anti-KLK2 and effectorless IgG Fc (Shang et al., 2014)	N = 165 (Estimated) mCRPC, refractory to at least either 1 line chemotherapy or novel androgen receptor targeted therapy, ECOG 0–1	s.c. ingection, s.c. infusion, or i.v. infusion, dosing not specified. (Author Anynomus, 2021)	Not reported	Not reported	Not reported	Recruiting, due primary completion November 2024	NCT04898634
JNJ-70218902 (JNJ-902)	1	Anti-CD3 and anti-TMEFF2	N = 82 mCRPC refractory to at least either 1 line chemotherapy or novel androgen receptor targeted therapy, ECOG 0–1	Not specified	45% fatigue, 44% anorexia, 37% injection related reaction, 33% anaemia, lower back pain 25%, arthralgia 22%	Reported as “uncommon” but not specified	PSA50 reduction in 8 subjects. PR in 5 subjects. (Calvo et al., 2022)	Active, no longer recruiting. Preliminary data released	NCT04397276
Tarlatamab (AMG 757, BI 764532)	1b	Anti-CD3 and anti-DLL3 and IgG1 Fc (HLE) (sequences not specified)	N = 41	i.v. route, dosing not specified	Pending	Pending	Pending	Active, no longer recruiting	NCT04702737
			*De novo* or treatment emergent NEPC refractory to ≥1 systemic therapy, ECOG 0–2 (Aggarwal et al., 2021)						
LAVA-1207	1	Anti-PSMA and anti- Vδ2 (of Vγ9Vδ2T cells) (Gammabody^®^ construct) (Sequences not specified)	N = 66 (Estimated) mCRPC, refractory to ≥1 taxane chemotherapy regimen and novel androgen-receptor targeting therapy, ECOG 0–1	i.v. fortnightly, dose ranging 1.5–40 µg	Nil Grade ≥3 to date	Not reported	SD in 3 of 8 subjects to date (8 weeks therapy) (Mehra et al., 2023)	Recruiting	NCT05369000
AMG 340 (TNB-585)	1	High affinity anti-PSMA and low affinity anti-CD3 and Fc domain (HLE)	N = 100 (estimated) mCRPC refractory to ≥2 systemic therapies, ECOG 0–2	3 weekly i.v. infusion	Pending	Pending	Reduced cytokine release but equivalent anti-tumour activity against PC cell lines *in vitro* compared with higher affinity anti-CD3 constructs	Active, no longer recruiting	NCT04740034
		(Sequences not specified) (Buelow et al., 2021)							
Xaluritamig (AMG 509) ± enzalutamide or abiraterone	1	Anti-CD3 and anti-STEAP-1 and efffectorless IgG1 Fc domain (HLE) (Sequences not specified) (Nolan-Stevaux et al., 2023)	N = 97 mCRPC refractory to ≥1 systemic therapy (>80% had received previous taxane based chemotherapy regimen), ECOG 0–1	Weekly or fortnightly i.v. target dose ranging 0.001–2.0 mg, with step-up dosing and dexamethasone pre-medication	72% any grade CRS, 69% Grade 1–2 CRS, 45% fatigue, 32% pyrexia. 55% Grade ≥3 AEs (anaemia 13%, myalgia 12%, fatigue 11%)	54% treatment emergent ADAs, 51% were neutralising, 45% reduced drug exposure >25%. However, ADAs not associated with PSA50 response at 12 weeks therapy, or with AEs	24% PR, 48% SD, 19% PD. 49% PSA50 reduction, 28% PSA90 reduction for all doses. (Kelly et al., 2023)	Recruiting. MTD identified = 1.5 mg i.v. weekly with 3 tier step-up dosing	NCT04221542
REGN4336 ± cemiplimab	1/2a	Anti-CD3 and anti-PSMA, limited details available on structure	N = TBC mCRPC refractory or intolerant to ≥2 lines systemic therapy and ADT	Weekly or 3 weekly s.c. injection, dosing not specified ± 3 weekly i.v. cemiplimab. (Kelly et al., 2022)	Pending	Pending	Pending	Recruiting	NCT05125016

Abbreviations: CD3, Cluster of differentiation 3, marker of T cells; PSMA, Prostate specific membrane antigen; mCRPC, Metastatic castrate resistant prostate cancer; ADT, Androgen deprivation therapy; ECOG, Eastern Cooperative Oncology Group Performance Status Scale; C.i.v., Continuous intravenous infusion; MTD, maximum tolerated dose; Fc, Crystallisable fragment; HLE, Half-life extended; PSCA, prostate stem cell antigen; KLK, human kallikrein; DLL3, Delta-like protein 3; NEPC, Neuroendocrine prostate cancer; STEAP-1, Six transmembrane epithelial antigen of prostate.

## 2 Discussion

### 2.1 Structural alterations to extend half-life

The original BiTE molecules were comprised of two single chain variable fragments (scFv) from two different mAbs connected by a flexible peptide linker (Ahmad et al., 2012). The small size of these molecules resulted in a short half-life with poor serum stability and rapid renal clearance, which necessitated administration via continuous intravenous infusion. This issue was subsequently addressed with the development of half-life extended (HLE) BiTE variants, which incorporated structures to increase molecular size and stability, enabling BiTEs to be formulated as intermittent infusions or subcutaneous injections, and consequently improving efficacy, convenience and cost of administration schedules. One method involved bonding together of two BiTEs to create a tetravalent “tandem diabody” (TandAbs; (Kipriyanov et al., 1999)). Alternative methods included addition of antibody heavy chain elements, either as part of a IgG-based structure, or as an isolated Fc domain to enable binding to the neonatal Fc receptor in recipient serum and thus reduce the rate of clearance (Brinkmann and Kontermann, 2017). Inclusion of an Fc region confers the ability to bind to the neonatal Fc receptor on innate immune cells, enhancing immune cell engagement. Other HLE strategies have included addition of an albumin receptor to improve serum stability, or molecular modification of heavy chains to increase half-life, such as with the XmAb technology (Zhukovsky et al., 2007). Overall, these customisations have created a myriad of BiTE structural variations which have facilitated administration via intermittent intravenous or subcutaneous routes, and also offer additional functional benefits.

### 2.2 Excessive adverse events and immunogenicity

#### 2.2.1 Class specific adverse events

The main adverse events of special interest with BiTEs are cytokine release syndrome (CRS) and immune effector cell-associated neurotoxicity syndrome (ICANS). CRS is a systemic inflammatory response syndrome that can produce symptoms ranging from fever to shock with multi-organ failure caused by high levels of cytokine release, particularly IFN-γ, IL-1 and IL-6 (Morris et al., 2022). CRS most commonly occurs hours to short days after the initial dose of TCE therapy, with some evidence showing reduced incidence and severity following pre-medication with dexamethasone and step-up dosing regimens (Shimabukuro-Vornhagen et al., 2018). Severe CRS can be treated with tocilizumab, an anti-IL-6 receptor mAb, without affecting therapeutic activity (Kauer et al., 2020). The occurrence of any-grade CRS in the studies on BiTE therapies in mCRPC to date ranges from 5% with JNJ-902% to 88% with CC-1, although it should be noted that there were no reports of associated mortality even with higher rates of CRS (Calvo et al., 2022; Heitmann et al., 2022).

ICANS pathophysiology remains uncertain, but patients often present with confusion, tremor or dizziness and can progress to seizures or irreversible encephalopathy over a course of days post treatment. Treatment consists of aggressive supportive therapy and corticosteroids. Tocilizumab is unable to cross the blood-brain barrier and has limited clinical utility in management. ICANS occurs infrequently with BiTE therapies in general and appears to be rarer in solid tumour BiTE studies (Siegler and Kenderian, 2020). There have been no formal diagnoses of ICANS in the studies of prostate cancer-targeted BiTEs to date, although seizures, a possible sign of ICANS, was reported in a single patient receiving HPN-424 (Bono et al., 2021).

CRS initially presented a clinical challenge to progression of BiTE therapies in solid tumours. However, with increased exposure to and recognition of the constellation of CRS in clinical settings, the improved availability of tocilizumab, and the growing evidence that even with high grade CRS, survival remains extremely high, this trAE should not be a major factor limiting use of BiTE therapies moving forward. Focus should instead be placed on reducing immunogenicity of the BiTE structure to curb hyperactive cytokine release where possible. Brandt et al. have proposed the “kill switch” mechanism for BiTE therapy similar to that adopted in experimental CAR T-cell therapies (Brandt et al., 2020). These mechanisms rely on the incorporation of an inducible self-destruct process into the original therapy which, when activated, results in the rapid removal of the treatment from the system. In CAR T-cell therapies, this has involved induction of the caspase-9 T-cell apoptosis pathway, or induction of T-cell transcription of viral components subsequently leading to T-cell apoptosis. While purely theoretical, inclusion of a cleavage site within the linker portion of a BiTE therapy could achieve similar effects.

#### 2.2.2 On-target off-tumour effects

A challenge in the adaptation of BiTEs from haematological to solid tumours has been the difficulty in finding solid organ TAAs which are highly specific to the desired tumour cell population, as many solid organ TAAs are also present at low levels in normal tissue. In this instance, BiTE activity poses a risk of so-called “on-target off-tumour” effects resulting from damage to these non-malignant tissues. These can be catastrophic, as demonstrated in trials of catumaxomab, an early example of a solid organ BiTE directed against epithelial cell adhesion molecule (EpCAM) and CD3 for EpCAM-positive advanced solid organ cancers causing malignant ascites, with one patient experiencing fatal acute fulminant liver failure (Linke et al., 2010).

PSMA, the most commonly targeted TAA in prostate cancer BiTE therapies to date, while being highly expressed in prostate cancer, also has high levels of expression within intestinal, liver, salivary glandular cells and proximal kidney tubules (Silver et al., 1997). There has been evidence of PSMA targeting leading to significant on-target off-tumour effects, for example, with the BiTE HPN424, which caused Grade 3–4 AST elevations in 18% patients, and ALT elevations in 11% (Bono et al., 2021). Although on-target off-tumour AEs appear to be relatively infrequent in trials of BiTEs targeting PSMA, research into more specific TAAs remains warranted to alleviate the risk of these outcomes.

#### 2.2.3 Alternative TAAs

Multiple alternative TAAs are under investigation as therapeutic targets in prostate cancer. One potential target proposed has been STEAP-1, which has some expression in lung tissue but otherwise has limited expression outside of the brain which is considered inaccessible to BiTE molecules (Xu et al., 2022). STEAP-1 has been targeted in a Phase 1 clinical trial using an agent called xaluritamig. Although 72% of patients experienced CRS, the vast majority (69%) were Grade 1–2 (Kelly et al., 2023). Another target being used in clinical trials is TMEFF2 which has expression limited to intestinal tissues, the male reproductive tissues and brain. JNJ-70218902 was designed to target TMEFF2 and entered phase I testing but limited available data exists regarding its efficacy and toxicity profile (Calvo et al., 2022). Kallikrein related peptidase-2 (KLK2) is a highly specific TAA with expression restricted to prostate tissue only and is the putative tumour target for JNJ-78278343. Both efficacy and safety data is still being awaited at this stage (Shang et al., 2014).

The current trials of BiTEs in prostate cancer treatment as described in Table 1 are all targeted at membrane bound extracellular proteins which can be readily accessed by nearby immune effector cells. However, these extracellular proteins represent only a fraction of potential tumour specific targets. Intracellular protein fragments expressed extracellularly bound to human leukocyte antigen (HLA) major histocompatibility complexes (MHC) through antigen presentation to immune cells offer a much more extensive suite of potential therapeutic targets not otherwise accessible to antibody based therapies (Trenevska et al., 2017). BiTEs designed to target these peptide-HLA complexes have been termed immune mobilising monoclonal T-cell receptors against cancer (ImmTACs). Tebentafusp, an approved BiTE therapy for uveal melanoma, is the first in class ImmTAC, designed to target a peptide-HLA combination of gp100:HLA-A*02:01 along with the CD3 component of the T-cell receptor (TCR, Nathan et al., 2021). Disadvantages of this therapy include restriction of eligibility of patients to those with matched HLA typing, challenges of identifying peptide:HLA combinations which are highly conserved across cancer cells and patient populations, and possibility of tumour evasion through downregulation of HLA cell surface expression (Trenevska et al., 2017; Maruta et al., 2019). Nevertheless, the development of ImmTACS represents a breakthrough in BiTE development, and further research into intracellular prostate cancer TAAs may reveal novel therapeutic targets with reduced on-target off-tumour effects.

#### 2.2.4 Increasing specificity of localisation of BiTE activation to tumour tissue

In addition to targeting more tumour-specific TAAs, another approach to improve the localisation of BiTE activation to tumour tissue involves the incorporation of moieties targeting two different TAAs into a single BiTE construct. This has been explored pre-clinically in solid tumour cell lines with AMG-305, a dual targeted BiTE directed against P-cadherin and mesothelin which showed attenuated activity against cells expressing only one of these targets (Pham et al., 2023). Targeting of two separate TAAs may also offer a possible means of reducing immune escape through the common route of downregulated expression of a targeted TAA. However, once again, in the absence of TAAs which are more tightly limited to prostate cancer expression, these therapies continue to pose the risk of unwanted on-target off-tumour effects.

Another possible strategy to localise BiTE activation to tumour tissue is the incorporation of structural elements which prevent activity of the BiTE outside of conditions specific to the tumour microenvironment. One possible approach is masking of BiTE binding sites with structures designed to be cleaved away by tumour-resident proteases, hence restricting activity to tumour deposits (Geiger et al., 2020). Panchal et al. described generation of an EGFR-CD3 BiTE where the heavy and light variable chains of the anti-CD3 portion were separated by a linker degradable only by the tumour specific matrix metalloproteinase 9 (MMP9), permitting anti-CD3 activity only after MMP9 facilitated rearrangement of the molecule (Panchal et al., 2020). Another approach is addition of a second TAA target within a T-cell engager structure with the aim of improving tumour tissue specific cytotoxicity. A variation on this concept is the construction of “hemibodies,” two “half” antibodies each containing a different antigen binding scFv fragment fused to either a variable light or variable heavy chain of a CD3 antibody, designed to recombine to form a functional BiTE only in the presence of both TAAs. The proof-of-concept hemibody was targeted against HLA-A2 and CD45, and showed apoptosis restricted to dual positive tumour cells in animal models (Banaszek et al., 2019). To date these experiments have all been pre-clinical, and none have been targeted towards prostate cancer, however these results could be transferable to other BiTE structures in future (Panchal et al., 2020).

#### 2.2.5 Immunogenicity and antidrug antibodies

Drug related immunogenicity, or the response of a patient’s immune system to a drug, occurs via recognition of drug components as “non-self,” and is a key factor influencing the efficacy and adverse effects of BiTEs (Jawa et al., 2020). Protein sequences within the drug or drug excipients as part of the drug formulation are taken up by patients’ antigen presenting cells (APCs), with subsequent breakdown and expression of short protein epitopes on HLA surface molecules. T helper cell recognition of these epitopes as “non-self” will stimulate an immune response including a B cell humoral response with production of anti-drug antibodies (ADAs). ADAs can be broadly split into “neutralizing” antibodies which obstruct binding sites, and “non-neutralizing,” which bind epitopes which do not directly interfere with the drug’s action. ADAs have critical effects on a drug’s efficacy, pharmacokinetics, and adverse events through a wide array of mechanisms, including blocking or changing affinity of binding sites, prolongation or potentiation of drug clearance, aggregation of drug-antibody complexes and effects from inflammatory cytokine production (van Brummelen et al., 2016). Further complicating factors influencing the extent of immunogenicity to a drug include the route of administration, dose regimen, product storage, product purity, and the patient’s own immune system factors such as the presence of pre-existing cross-reacting ADAs and skew towards immunoregulatory or inflammatory immune responses. The desired alternative response is recognition of these epitopes as “self” by the immune system, with subsequent induction of immune tolerance via activation of T regulatory cells (Tregs).

The complex interactions between these therapies and the immune system are a fundamental part of the nature of T cell therapies. ADA quantification and their resultant physiological effects form the backbone of a multifaceted immune response assessment recommended by both the FDA and EMA for drug immunogenicity (Administration USFaD, 2010; Agency, 2017). It should be noted that current detection methods for ADAs are imperfect. A range of assays can be used to detect ADAs, with a wide variability in sensitivity and specificity. Furthermore, assays usually only detect one subclass of Fc immunoglobulin receptors, predominantly IgG, and may not detect drug-bound antibody, leading to under-reporting of ADAs (van Brummelen et al., 2016). Nevertheless, ADA analysis remains the most accurate method of assessing immunogenicity. Notably, these assessments are primarily conducted *in* or *ex vivo* in human clinical studies, given the limitations to mimick a nautral human immune response in animal or human cell lines.

Where data has been released for quantification of ADAs in clinical trials of BiTEs for prostate cancer, ADAs were frequently present in >50% subjects (See Table 1). This is an extremely high incidence compared to other biologic therapies on the market (van Brummelen et al., 2016). When compared with intravenous administration, subcutaneous dosing is associated with a high rate of neutralising antibodies, which has been ascribed to sequential antigen presentation from both APCs residing in the skin and then lymph node-resident APCs. These two “waves” of antigen presentation increase the formation of ADAs (Jarvi and Balu-Iyer, 2021). For instance, during testing of pasotuxizumab, induced ADAs were recorded in 100% subjects receiving s.c. dosing, with 93% of these being neutralizing, but none in those receiving the continuous i.v. dosing (Hweixian et al., 2023).

Further assessment of ADA subclasses and effects in BiTE therapies for mCRPC has been limited to studies in pasotuxizumab and xaluritamig (Hweixian et al., 2023; Kelly et al., 2023). Within this limited sample, it has been demonstrated that treatment emergent neutralizing ADAs can have either adverse (e.g., pasotuxizumab) or neutral (e.g., xaluritmag) clinical effects. (Hweixian et al., 2023; Kelly et al., 2023). For xaluritamag, 54% of subjects developed treatment emergent ADAs for an i.v. formulation, with 45% of subjects developing neutralising antibodies causing reduction in drug exposure by more than 25%. However, these ADAs were not associated with difference in PSA50 response at 12 weeks (Kelly et al., 2023). Conversely, non-neutralising ADAs can lead to formation of serum antibody complexes which are subsequently removed by the host immune system, which theoretically could affect drug efficacy despite their “benign” status, although this was not observed in the above two trials. Despite historical data in other fields showing an association between ADAs and certain AEs, the presence or titre of ADAs does not appear to correlate with the rate or severity of AEs in either population. Importantly, all ADAs, regardless of neutralising status, can dramatically alter pharmacokinetics of the drug by sustaining or expediting drug clearance, with likely construct specific effects on efficacy (Kelly et al., 2023). Unfortunately, a deeper analysis is limited by the restricted published data from clinical trials about ADAs and their effect on drug pharmacokinetics or pharmacodynamics.

The causes of the high rates of ADA formation in response to BiTE therapies are incompletely understood but derives at least partially from particularly immunogenic sequences within the drug’s structure, including effector Fv domains, Fc region, half-life extending domains, or peptide linkers. A number of methods can be used to determine the immunogenicity of these sequences, including screening of drug protein structure for known T helper or T regulatory binding epitopes; culturing of antigen presenting cells with *ex vivo* peripheral red blood cells with sequencing of epitopes expressed by APCs; or analysis of the binding sequences of extracted ADAs from trial subjects to match to a complementary epitope from the drug; with the latter detailed by Hweixian et al. for pasotuxizumab (van Brummelen et al., 2016; Hweixian et al., 2023). However, the protein sequences of the drug or ADAs detected have not been made available to the public domain for the majority of BiTEs trialled for prostate cancer so far. Release of this existing information, and wider testing of ADAs in ongoing and future trials would provide invaluable information in helping better understand the interplay between BiTEs and the humoral immune system. It could help to differentiate the concentrations or affinities at which ADAs become clinically significant, and to select drug components with less problematic immune responses. With this information, immune engineering of therapeutic protein structures could enable adjustments to homology to mask or remove immunogenic sequences, or addition of T regulatory epitopes promoting immune tolerance (van Brummelen et al., 2016).

Other factors known to contribute to immunogenicity include drug excipients, which can be comprised of contaminant proteins accidently purified with the therapeutic protein during production, or other components of the drug formulation such as trace heavy metals. Subcutaneous administration has been associated with increased ADAs over an i.v. route for previous biologics, as has re-exposure following a treatment free interval (Agency, 2017). Half-life extending domains, such as an Fc region, may also add to non-humoral immunogenicity through engagement of the immune system via the Fc receptor on APCs, neutrophils and NK cells. Although HLE molecules are attractive for the possibility they offer of a more convenient administration schedule, the added immunogenicity they seem to generate poses challenges, particularly to subcutaneous administration. Finally, there are patient specific factors at play, such as levels of pre-existing ADAs, and different HLA haplotypes influencing the particular epitopes that may produce a patient response (van Brummelen et al., 2016). Once again, limited testing or release of data concerning ADA quantity and sequences, and their association with patient HLA typing and drug excipients, prevents retrospective analysis of how to avoid highly immunogenic structures in the future. Greater transparency and dissemination of available data will assist in further targeted development of this class of agents.

### 2.3 Limited anti-cancer effect

Unfortunately, the majority of completed clinical trials of BiTEs against prostate cancer have demonstrated limited or inconsistent anti-tumour effects to date. There is a recurrent pattern of small percentages of study groups achieving PSA50, or radiologic partial response (PR) in clinical trials examining the use of HPN424, JNJ-081 and JNJ-902 (Bono et al., 2021; Calvo et al., 2022; Lim et al., 2022). Pasotuxizumab displayed slightly more promising results with a 19% PSA50 response and two long term PSA50 responders, however this trial was prematurely terminated in favour of acapatamab, which in turn delivered a PSA50 response in 34.3% of subjects (Ben et al., 2020; Hummel et al., 2021). However, the trial using acapatamab was also discontinued in the setting of a high incidence of trAEs. Xaluritamig has recently demonstrated the greatest anti-tumour efficacy in the field, with 49% of patients demonstrating a PSA50 response and 28% of patients also achieving a PSA90 reduction (Kelly et al., 2023).

Despite these promising results, when compared with haematological malignancies, immunotherapies in prostate cancer face multiple barriers to effectiveness, including intra- and inter-tumoral genotypic heterogeneity, and downregulation of TAA expression over time, leading to treatment escape or failure (Middelburg et al., 2021; Ge et al., 2022). They must also contend with a complex solid tumour microenvironment (TME), of which prostate cancer’s TME presents specific challenges.

#### 2.3.1 Immunosuppressive “cold” tumour microenvironment

The disappointing response of prostate cancer to ICIs has been predominantly attributed to its multifactorial “cold” or immunosuppressive tumour microenvironment (TME) in comparison to other common malignancies such as melanoma or lung. Metastatic CRPC TMEs are characterised by a dense stroma with relatively high proportions of cancer-associated fibroblasts (CAFs) and myeloid derived suppressor cells (MDSCs) which presents a physical barrier to anti-cancer therapies and results in low numbers of tumour infiltrating lymphocytes (TILs) and innate immune cells. Even within the limited TIL population there is a tendency towards an immunosuppressive phenotype, with a preponderance of Th2 and T regulatory (Treg) lymphocytes over Th1 counterparts, skewing against activation of CD8^+^ cytotoxic T cells and natural killer (NK) cells (Krueger et al., 2019). There is a similar overabundance of M2 anti-inflammatory macrophages instead of their inflammatory M1 counterparts. Metastatic CRPC TMEs are further masked from the immune system by a low tumour mutational burden with consequent reduced neoantigen expression as well as local overexpression of costimulatory molecules such as PD-1 and CTLA-4, which act as “self” markers, leading over time to an “exhausted” local immune cell phenotype (Gannon et al., 2009).

This immunosuppressive TME is theorised to limit the efficacy of the “bystander” effect of immunotherapies, in which successful cell dependent cytotoxicity creates a local environment of pro-inflammatory cytokines and subsequent upregulation of neoantigens and differentiation of inflammatory immune cell phenotypes, prompting cytotoxicity towards adjacent tumour cells (Ross et al., 2017). Notably, ADT, the cornerstone of prostate cancer treatment, is suspected to contribute to the immunosuppressive TME through increases in intratumoural Tregs, MDSCs and M2 macrophages, reduced markers of interaction between tumour cells and de-regulation of intratumoural Tregs (Pu et al., 2016; Qin et al., 2022). The immunosuppressive TME of prostate cancer is observed to become more extreme with progression to mCRPC, with bony metastases representing the most severe example of this phenotype (Jiao et al., 2019). Synchronous metastatic deposits of CRPC display significant genetic heterogeneity, further predisposing to immune escape and treatment resistance (Sun, 2021).

BiTEs were initially expected to provide a radical solution to many of the problems presented by “cold” TMEs, by localising T cell activation directly to malignant cells. Unfortunately, this has not eventuated, with BiTE therapies demonstrating limited anti-tumour activity in prostate cancer in studies to date. It is possible that the immunosuppressive TME limits the bystander effect of BiTEs to some extent, as it does for ICIs. It should also be noted that the recipients of these BiTEs comprise a heavily pre-treated and castrate-resistant group. In light of the mCRPC TME being highly immunosuppressive post ADT, ARSi and chemotherapy, it can be speculated that BiTEs may show greater efficacy in a less heavily pre-treated patient population, akin to the benefit seen in a similar sub-population with the sipuleucal-T vaccine.

#### 2.3.2 Mitigating the mCRPC TME

There are a multitude of theories as to how to alter the TME pathophysiology to improve the action of BiTEs, predominantly based on improving either systemic or intratumoural immune effector cell populations and activity. A logical option is to trial a combination of immunotherapy with BiTE therapy, to simultaneously aim to reverse TIL anergy, prevent BiTE mediated upregulation of TIL PD-1 expression and tumour and stromal PD-L1 expression (Jiang et al., 2019; Belmontes et al., 2021). This strategy has been tested in prostate cancer BiTE trials (See Table 1), with combinations of anti-PSMA BiTEs with PD-1 inhibitors, namely, acapatamab and pembrolizumab, and REGN4336 and cemiplimab, but results from these combinations are not available (Ben et al., 2020; Kelly et al., 2022). Outside of prostate cancer, there has been great interest in this combination with multiple clinical trials underway (Belmontes et al., 2021). In solid tumours, preliminary phase 1 clinical trial data for a CEA and CD3 targeting BiTE showed increased disease response when paired with atezolizumab in the absence of increased toxicity (Tabernero et al., 2017). Alternative structural variations on the synergy of BiTEs and ICIs includes bifunctional checkpoint-inhibitory T-cell engagers (CiTEs), comprised of a BiTE crosslinked with a PD-L1 inhibitor to provide localised combination therapy and attempt to avoid systemic effects of ICIs (Herrmann et al., 2018).

An alternative experimental approach involves shifting focus to disruption of the stroma of the TME to permit increased TIL migration and improve BiTE intra-tumoural access. Brunker et al. developed a bi-specific antibody designed to crosslink the fibroblast activation protein (FAP) and the death receptor 5 (DR5), triggering the extrinsic apoptotic pathway for tumour cells, with successful cytotoxicity in FAP positive tumour stroma (Brünker et al., 2016). Another example of successful pre-clinical alteration of the structure of the TME has been the use of an oncolytic virus expressing a FAP-CD3 BiTE which successfully increased intra-tumoral accumulation of T cells and decreased FAP concentration, indicating fibroblast apoptosis *in vivo* testing (de Sostoa et al., 2019). The extracellular matrix could also be directly targeted with enzymes such as hyaluronidase to literally open a path for tumour infiltration by T cells (Eikenes et al., 2005). Yet further studies have targeted function and trafficking of MDSCs to reduce their immunosuppressive local effects (Middelburg et al., 2021). However, these approaches remain pre-clinical and have not been targeted to prostate cancer to date.

#### 2.3.3 Alternative immune cell targets

Rather than focusing on CD3 positive T-cells, some potential therapeutics instead aim to target alternative immune effector cell populations such as the pro-inflammatory natural killer (NK) cells. NK cells have been targeted via bi- and tri-specific NK-cell engagers (BiKEs and TriKEs) in clinical trials for haematological malignancies with promising results (Rothe et al., 2015; Vallera et al., 2016). γδ T-cells present a unique target in prostate cancer. Although they are relatively sparse in comparison to the more common αβ T-cells, they are particularly concentrated in prostate cancer tumours when compared with other solid organ tumours (Tosolini et al., 2017). Activation of intra-tumoral γδ T-cells could potentially kickstart immune cell activation within the prostate TME, and a clinical trial of LAVA-1207, a γδ T-cell-directed BiTE, is currently underway (Mehra et al., 2023).

#### 2.3.4 Increased binding efficacy

A potential strategy for overcoming limited anti-tumour activity of current BiTEs would be to increase the binding affinity of the TAA or CD3 targeted structural components. Multiple antibody components targeting the same TAAs could be added to increase valency and target binding, as exemplified by the TandMab structure. Alternatively, reverse protein engineering could be utilised to design increased affinity of complementarity determining regions within the variable chains directed towards the relevant TAA. For example, Zekri et al. (2021) developed a proprietary PSMA binder, 10B3, which demonstrated increased reactivity against prostate cancer cells *in vitro* compared with a pre-existing J591 PSMA antibody. This improvement was attributed to alternative binding sites to PSMA recognised by the 10B3 molecule. However, it should be noted that protein engineering generally leads to deviation from native antibody structure and consequently higher risk of immunogenicity. Ultimately, while the expression of BiTE targets for prostate cancer remains non-specific to malignant tissue, attempts to increase TAA binding affinity risk increased rates of on-target off-tumour effects.

## 3 Conclusion

Metastatic CRPC is a prevalent disease which remains a challenging clinical entity to effectively treat, with limited response to the ICIs which have become cornerstones of treatment for multiple other cancers. Novel TCE therapy, particularly BiTE therapy, has been a promising area of immunotherapy development in the past decade with particular interest in their use in prostate cancer. Unfortunately, early investigations of various prostate cancer specific BiTE therapies have been limited by high incidences of intolerable adverse events and insufficient anti-tumour activity. Nevertheless, progress has been made in addressing these shortcomings through identifying a range of possible TAAs to exploit, extending the drug half-life allowing for more convenient administration schedules, and managing class specific AEs. Moreover, there is extensive research into multiple strategies as to how to overcome the challenges presented by the prostate cancer TME, the immunogenicity of BiTE constructs and focused targeting of BiTEs to tumour cells. With these developments and the possibilities of identification of more specific TAAs, improved affinity of BiTEs to TAAs, greater modelling and testing for immunogenicity, and modulation or mitigation of the anti-inflammatory mCRPC TME, it is reasonable to hope that BiTEs may provide a therapeutic benefit in the future for those afflicted with mCRPC.
